# Potential Cost-Effectiveness of Machine Learning-Enabled Primary Care Identification of Hepatitis C Virus Patients in the US

**DOI:** 10.3390/v18030299

**Published:** 2026-02-28

**Authors:** Thomas C. S. Martin, Jeremiah Wilson, Ashley Pitcher, Jessica Frankeberger, Susan J. Little, Natasha K. Martin

**Affiliations:** 1 Division of Infectious Diseases and Global Public Health, University of California San Diego, La Jolla, CA 92093, USA; 2 IQVIA, Copenhagen 2200, Denmark; 3 Edson College of Nursing and Health Innovation, Arizona State University, Phoenix, AZ 85004, USA

**Keywords:** HCV, elimination, machine learning, screening

## Abstract

Machine learning (ML) algorithms may be effective at improving the HCV care cascade. One ML algorithm, developed using U.S. ambulatory electronic medical records (EMR), demonstrated the ability to identify people infected with HCV earlier than conventional testing strategies among those with indications for screening. We evaluated the potential cost-effectiveness of ML-enabled screening for the early identification of undiagnosed HCV among people in care in the U.S. An HCV natural history Markov model was developed to evaluate the cost-effectiveness of the ML algorithm-enabled screening compared to conventional testing over the training data period. Based on the training data, the ML algorithm identified patients on average 6.5 months earlier than conventional testing strategies. We compared the status quo to intervention scenarios using the ML algorithm at different recall levels (proportion of HCV patients identified, 5–100%). We identified the optimal algorithm recall level, which maximized health (measured in quality-adjusted life years, QALYs) while staying under a willingness-to-pay threshold of USD$100,000/QALY gained. ML-enabled screening was cost-effective (ICER < $100 k/QALY gained) in identifying undiagnosed HCV patients for recall levels up to 30%. The optimal recall level was 30% (Precision 0.27%), which resulted in a mean ICER of $94,022/QALY gained. ML-enabled screening for the early identification of undiagnosed HCV patients could be cost-effective in the U.S. Prospective evaluation of real-world effectiveness is warranted.

## 1. Introduction

Untreated hepatitis C virus (HCV) infection is a substantial health burden, with more than 2 million people estimated to be currently infected in the U.S. and billions of dollars spent on sequelae each year [1,2]. Despite new recommendations by the Centers for Disease Control (CDC) and U.S. Preventative Services Taskforce for one-time universal hepatitis C virus (HCV) screening of all adults ages 18 or older [3], many individuals remain undiagnosed in the United States [4], even more so due to the COVID-19 pandemic [5]. Direct-acting antiviral treatment for HCV is highly effective (>95% cure), but as many patients remain undiagnosed, many remain untreated.

Screening guided by machine learning (ML) algorithms is potentially an effective way to improve HCV diagnosis rates. Such algorithms have already been developed for diagnosing patients with Alzheimer’s and type 2 diabetes with promising results [6,7]. In addition, one recent ML algorithm trained using patient claims data in the United States proved to be highly effective at predicting HCV status [8]. Although the algorithms in those studies differed in application, an accurate ML algorithm to assist with the identification of people with undiagnosed HCV could be cost-effective.

One ML algorithm, developed using U.S. ambulatory EMRs with a retrospective training and validation dataset between 2015 and 2020, showed that among individuals with an indication for HCV testing, it was able to identify people infected with HCV an average of 6.5 months earlier than conventional strategies [9]. Additionally, the authors found that an ML-guided strategy could yield higher precision compared to a risk-based or universal screening strategy [9]. By changing the algorithm specifications, the model could be adjusted by the user to prefer higher recall (sensitivity) or specificity, depending on the user’s needs. The ML algorithm performance was assessed for a range of recall levels to determine the corresponding precision values (positive predictive value), which ranged from 0.02 to 2% depending on recall [9]. However, the potential cost-effectiveness of adding an ML algorithm for earlier identification of undiagnosed individuals on top of current clinical workflows is unknown, and, if implemented, the optimal algorithm recall level is also unknown. Our analysis, therefore, aimed to model the potential cost-effectiveness of using such an ML algorithm to enable early identification of undiagnosed persons with HCV in the United States, and evaluated optimal algorithmic recall level.

## 2. Materials and Methods

**Overview:** We estimated the cost-effectiveness of HCV screening using an ML algorithm compared to standard of care screening among primary care patients in the United States. We adopted a health-care payer perspective and a lifetime time-horizon. We analyzed the potential benefits an ML algorithm can bring by diagnosing persons with HCV earlier than they would be diagnosed through current screening practices. We also examined the tradeoff between algorithm recall level and precision and observed how cost-effectiveness analyses can inform this decision.

**Machine Learning Algorithm:** Details of the ML algorithm and performance have been previously published [9]. The model was trained using 16 million patients with U.S. ambulatory EMR data across primary and specialty care from 2015 to 2020. The algorithm was developed to identify undiagnosed HCV patients in a 12-month prediction window using medical history from a 24-month lookback with a 1-month offset. The ML algorithm, using gradient boosted trees, was used to predict HCV vs. no diagnosis of HCV. On average, the algorithm identified patients with HCV infection between 1 and 12 months (~6.5 months on average) before they were diagnosed by conventional screening (personal communication).

**Figure 1:**
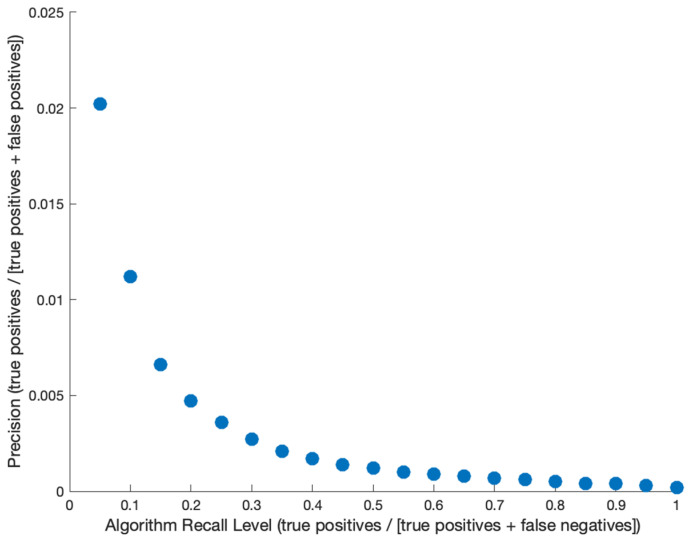
Relationship between ML algorithm recall level (sensitivity) and precision (positive predictive value).

**Intervention and Comparator:** Our intervention simulated the use of a machine learning algorithm for the early identification of undiagnosed HCV among patients in primary care in the United States, compared to screening recommendations at that time. Based on the algorithm’s performance, we assume the algorithm leads to early identification of patients with HCV infection (6.5 months on average), which we assume leads to earlier uptake of HCV treatment and therefore prevention of liver disease progression and associated costs. We determine the status quo of HCV detection rate in primary care from the EMR data (0.02% of individuals diagnosed in a 12-month period [9], Table 1), with demographic distributions of those detected in Table S1. We evaluated several comparator scenarios of using the algorithm at varying levels of recall (sensitivity) and precision (positive predictive value), shown in Figure 1. A higher recall means that more individuals are identified early (leading to prevention of liver disease progression), but that is also associated with lower precision (leading to higher costs related to testing individuals who do not have HCV). The recall and precision level that provided the highest health utility (measured in Quality Adjusted Life-Years [QALYs]) while staying under a willingness-to-pay threshold of $100,000 (USD) was considered the most cost-effective strategy [10].

**Figure 2:**
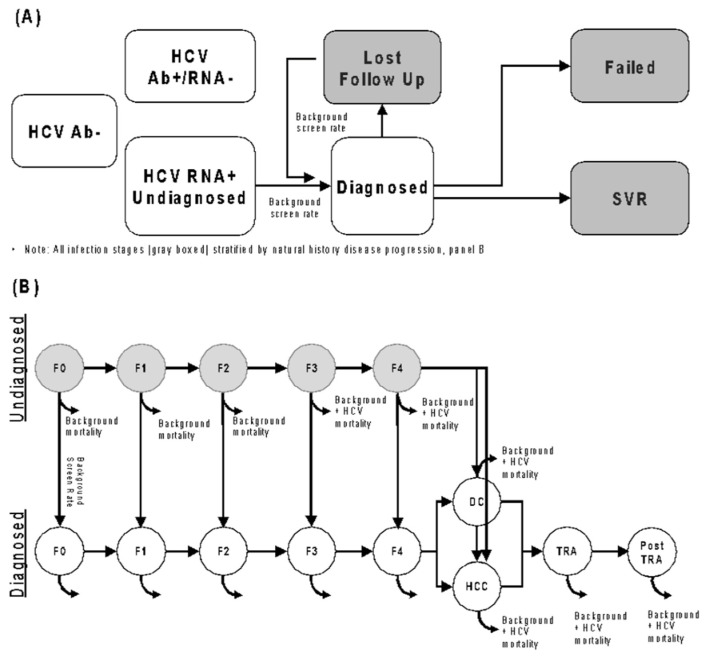
Diagram of the Markov model used in this analysis for the (**A**) HCV diagnosis and treatment cascade and (**B**) Disease progression cascade. HCV: hepatitis C virus. SVR: sustained virological response. Ab: antibody. HCC: hepatocellular carcinoma. DC: decompensated cirrhosis. TRA: transplant. RNA: ribonucleic acid.

**Model:** A closed-cohort, lifetime horizon Markov model (Figure 2) of HCV disease progression and treatment was used to estimate long-term health outcomes and costs, with additional costs of testing individuals without HCV also tracked in our analysis. The model tracked a fixed, theoretical population of 1 million patients over 100 years with demographics (age/sex/race/ethnicity/injecting drug use status) derived from the same ambulatory EMR data used to train the machine learning algorithm. “Per person” calculations use the initial population size of 1 million to determine per-person outcomes. The mixed-age population was stratified by HCV disease stage (METAVIR), diagnosis, and lost to follow-up status. The model specified that people lost to follow-up would need to be retested and diagnosed. Within the model, it was assumed that people who developed decompensated cirrhosis or hepatocellular carcinoma would be diagnosed upon progression to that disease stage. We assumed that, upon achieving sustained virological response (SVR), people with F3 stage disease or lower would not progress further, and that people with F4 or higher would progress at a slower rate. We did not incorporate retreatment or reinfection in our model.

**Model Parameters:** All model parameters and their corresponding sampling distributions can be found in Table 1.

Initial Cohort Characteristics: The initial theoretical population comprised 1 million individuals who were assumed to be in contact with primary care in the United States. Based on the EMR training population, 0.02% of these individuals would be newly diagnosed in the 11-month prediction window without additional intervention (equating to a 0.02% rate of new diagnosis in the one-year prediction period). Based on the training EMR data, the average age of the theoretical HCV population was assigned to be 50 years of age. The proportion of newly diagnosed HCV persons who were persons who injected drugs (PWID), part of the ‘birth-cohort’ of individuals born between 1945 and 1965, or the general population, changed with the recall level, can be found in Table 1.

Initial Fibrosis Distribution: Due to a lack of complete fibrosis stage data at diagnosis, the initial fibrosis distribution of the simulated HCV population was estimated using an HCV disease progression sub-model based on the observed age at diagnosis (stratified into 3 age categories: 0–34, 35–54, and 55+ years old). For each age group, we assumed the average age of HCV infection was 20 years old and simulated disease progression from the start of infection until the midpoint of each age group to determine the distribution of individuals within that age group across each disease stage. Using data on the age distribution of persons diagnosed with HCV for each algorithm recall level (Table S2), we were able to generate the estimated population fibrosis distributions for each recall level (Tables S3–S5).

Health State Parameters: QALYs and costs (USD) were assigned to each health state and a discount rate of 3% per year was used. QALY values and costs for health states were gathered from the published literature (See Table 1). A 0.05 increase in health utilities upon SVR was assumed, which is in agreement with similar analyses [38].

Transition Probabilities: HCV patients were stratified by METAVIR stage. Fibrosis progression rates were time-variant and obtained from a published study [11]. Background mortality rates were obtained from the WHO life tables (Table S6 [39]).

HCV Treatment Effect and Cost: We assumed a conservative 95% SVR rate across all genotypes consistent with other literature [20] and a cost of $25,000 per treatment course based on the 2025 Federal Supply Schedule [33,34].

HCV Testing Costs: Various testing costs were incorporated for all individuals. The testing yield in the status quo scenario was unknown, so it was calculated based on national estimates assuming a 51% HCV antibody prevalence for the PWID population [21] and a 1.6% prevalence for the birth-cohort population [23]. For the ML intervention, we included the cost of additional tests based on the recall and precision of the algorithm. We assumed a testing strategy for HCV antibody screening followed by HCV RNA PCR for those who screened positive. It was assumed that FibroScan would be performed on all individuals who test positive on HCV RNA PCR. Costs for these tests can be found in Table 1.

**Cost-Effectiveness Analysis:** Cost effectiveness in this analysis is measured in U.S. dollars (2025$) per QALY gained. We calculate the mean incremental cost-effectiveness ratio (mean ICER), calculated by taking the ratio of the mean incremental cost divided by the mean incremental QALYs between the baseline and comparator scenarios.

Probabilistic Multivariate Uncertainty Analysis: To account for variability in our parameters, a probabilistic uncertainty analysis was performed. Each parameter in the model was sampled from a unique sampling distribution (Given in Table 1) based on available research. A total of 1000 parameter sets were generated, and the model was run for each set of parameters. For each of 1000 runs of the model, we used the baseline and intervention costs and QALYs to calculate the incremental costs and QALYs gained and generated an ICER. We then took the mean of the incremental costs/QALYs from the 1000 simulations and the 2.5% and 97.5% percentile values to give a 95% confidence interval. An analysis of variance (ANOVA) was performed on the 45% recall level scenario to determine which parameters had the highest impact on our outcome variables (incremental cost, incremental QALYs, and ICER).

Univariate Sensitivity Analyses: Several one-way sensitivity analyses were performed to test model sensitivity to the following parameters: loss to follow-up rate, discount rate, time horizon, SVR, ML-enabled screening uptake, and predictive power of the algorithm. For the purposes of the parameter sensitivity analysis, we used the highest recall level for the algorithm under the $100,000 willingness-to-pay threshold as our base case (40% recall). We used a conservative 50% lost to follow-up rate in our original simulation and in our sensitivity analysis, we investigated whether using lower values (25% and 0%) had any effect on cost-effectiveness. We assess discount rates of 0% and 6% (compared to 3% at baseline). We also tested different values for HCV prevalence among the 1945–1965 birth-cohort and PWID. At baseline, we do not include a cost associated with the algorithm (although do include screening costs related to algorithm flags). For a sensitivity analysis, we determine the maximum algorithm cost where the algorithm would remain cost-effective using a 45% recall. Additionally, we examined time horizons of shorter length (25 and 75 years, compared to 100 years at baseline), lower SVR rate (90%, compared to 95% at baseline), and lower uptake of screening after ML algorithm identification (80%, compared to 100%).

Universal Screening Precision: Despite changes in HCV testing policy in 2020 to recommend a one-time screen of all adults aged 18–29, we lack data on the performance of the ML algorithm in the universal screening era. ML-enabled screening is likely to identify people with HCV earlier due to more targeted testing compared to universal testing; however, it is likely that some degree of individualized screening does occur even in the era of universal screening, which would reduce the impact of ML. We therefore assessed cost-effectiveness if the incremental impact of the ML algorithm was reduced from 6.5 months to 5 months to account for some degree of individualized screening within the universal screening era.

## 3. Results

**Figure 3:**
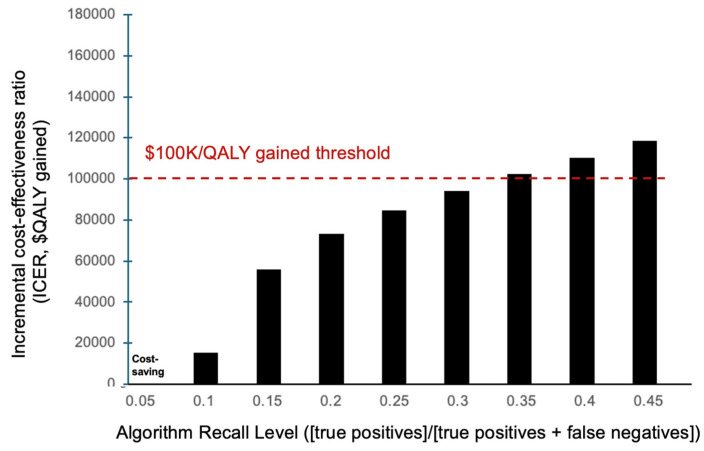
Incremental cost-effectiveness ratio (ICER, in $ per QALY gained) of using the machine learning algorithm to diagnose HCV patients compared to conventional testing practices for various recall levels. Red dashed line indicates the $100,000 willingness-to-pay threshold. ICER: incremental cost-effectiveness ratio, QALY: quality adjusted life-year.

The ML algorithm was found to be cost-effective with recall levels up to 30% compared to status quo screening (Figure 3). At 30% recall, the algorithm required additional per-person costs of $1.20 [95% CI: $1.07, $1.31], yielding a 0.000013 [95% CI: 0.000010, 0.000016] increase in QALYs per person over conventional testing practices. This led to a mean ICER of $94,022 per QALY gained (Table 2). The algorithm was potentially cost-saving for recall levels below 5% (Figure 3).

**Figure 4:**
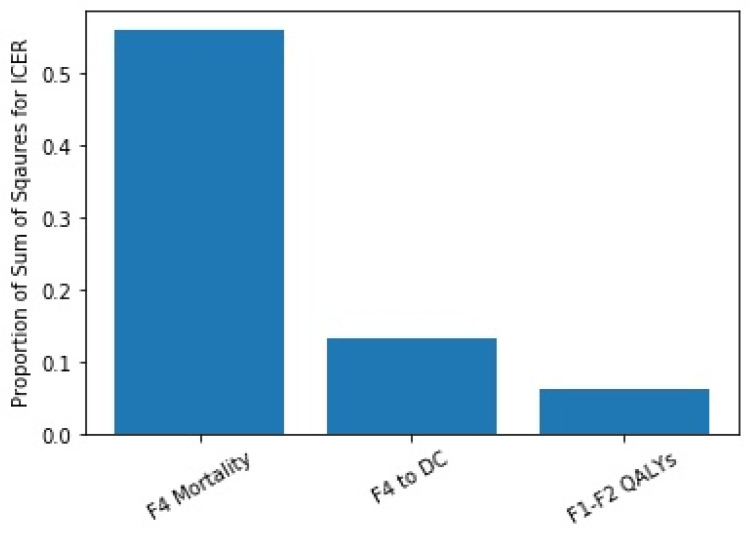
Results of the ANCOVA analysis on the ICER. DC: decompensated cirrhosis. QALYs: quality adjusted life-years.

The analysis of variance indicated that parameters associated with more advanced liver disease (F4 mortality rate, progression from F4 to decompensated cirrhosis, etc.) had the highest impact on our outcome variables (Figure 4). This is likely due to the age and fibrosis distribution of the simulated population modeled.

**Figure 5:**
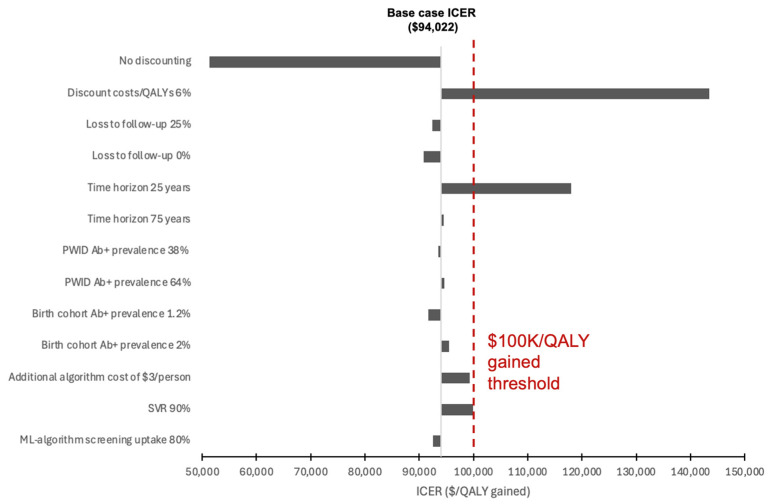
Tornado plot of univariate sensitivity analyses. The red dashed line represents a $100,000/QALY gained willingness to pay threshold. PWID: people who inject drugs. ICER: incremental cost-effectiveness ratio. QALY: quality adjusted life-year.

**Figure 6:**
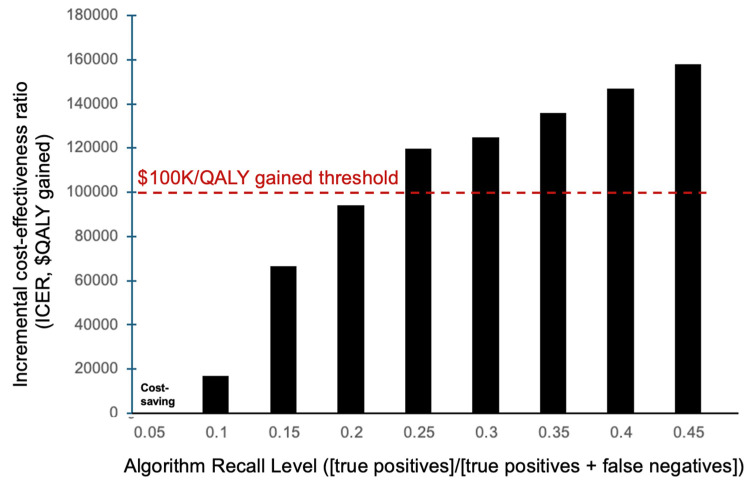
Sensitivity analysis of cost-effectiveness for each algorithmic recall level assuming the algorithm flags patients 5 months earlier (compared to 6.5 months in the main analysis). The red dashed line represents a $100,000/QALY gained willingness to pay threshold. ICER: incremental cost-effectiveness ratio, QALY: quality adjusted life-year.

The sensitivity analysis performed on the 30% recall level simulations indicated that the model’s cost-effectiveness results were minimally affected by variation in lost to follow-up rate (Figure 5). The ICER was sensitive to discount rate: a 6% discount rate increased the ICER over the willingness-to-pay threshold ($143,440 per QALY gained). A 25% relative difference in HCV antibody prevalence among PWID and the 1945–1965 birth cohort had minimal impact, remaining under the willingness-to-pay threshold. A shorter time horizon of 25 years decreased the cost-effectiveness ($118,010 per QALY gained). The model was insensitive to changes in loss to follow-up rates, provided loss to follow-up through ML detection was similar to conventional testing, with lower lost to follow-up rates associated with greater benefit of any screening but similar relative benefits of different screening strategies (ML vs. status quo). The intervention remained cost-effective (ICER < $100,000 per QALY gained) with lower SVR rates (90%) and reduced screening uptake after ML-identification (80%). The model remained cost-effective at 30% recall with a maximum cost of $3 per person flagged by the algorithm. The model was sensitive to changes in the incremental time to diagnosis with the algorithm. If the algorithm identified patients 5 months earlier (compared to 6.5 months at a baseline), the cost-effectiveness at a 30% recall was reduced to $136,005 per QALY (Figure 6) but remained cost-effective at algorithm recall levels of 20% or lower.

## 4. Discussion

Our model suggests that ML-guided HCV screening in ambulatory care in the United States is cost-effective for recall levels of up to 30% and potentially cost-saving at recall levels below 5%. Further, the algorithm would remain cost-effective at 30% recall with a maximum cost of $3 per person flagged by the algorithm. Our results add to the growing collection of literature indicating the utility of applying ML algorithms to healthcare data and supplement several HCV cost-effectiveness analyses spanning from risk-based to universal screening methods [25,32].

To our knowledge, this is the first analysis to apply the outcomes of an ML algorithm to identify undiagnosed HCV patients in a cost-effectiveness model and provides robust initial evidence to support continued evaluation of the ML approach. Our approach focuses on the cost-effectiveness of earlier diagnosis of people with HCV infection. However, there are likely to be additional advantages associated with ML-guided screening that are not captured in this study. Uptake of screening recommendations (risk-based, birth cohort, universal screening) has been limited in general–potentially due to issues such as lack of provider awareness, insurance barriers and patient access issues [40]. Use of an accurate ML algorithm may help automate this process or alert providers to individuals with a high probability of HCV infection, thereby improving diagnosis rates and reducing unnecessary testing for low-risk individuals. Improving the precision and specificity of testing algorithms would likely be cost-saving at all recall levels. Prospective trials are needed to validate the findings of HCV screening ML algorithms where reported precision is variable [8,9].

As with all cost-effectiveness analyses, there are limitations to our model. First, the ML algorithm was trained on retrospective data, thus we can only give an estimate of potential cost-effectiveness. The use of retrospective data means we could not account for patients who may be flagged by this algorithm that would otherwise be missed by conventional testing practices. Including this aspect would likely improve the cost-effectiveness of an ML algorithm. Work is underway to evaluate the ML algorithm on prospective data, which may provide further support for its cost-effectiveness. Second, there is uncertainty in the care cascade and current screening practices, which we explored through extensive sensitivity analyses. We found that our results are robust to uncertainty in some parameters, such as loss to follow-up rates after diagnosis, provided these are the same after diagnosis through ML screening or status quo screening. We believe it is reasonable to assume the care cascade after diagnosis would be similar, but further research is warranted. However, we note that the algorithm was primarily trained on data prior to universal screening recommendations in 2020, and therefore, the current performance is unknown. Our sensitivity analysis on the ‘lead time’ advantage with the ML algorithm indicated that although the incremental lead-time benefit of ML-enabled screening may be attenuated in the era of universal adult HCV screening, this does not inherently preclude cost-effectiveness. Rather, reduced lead-time gains shift the value proposition toward higher algorithm precision, with fewer false-positive identifications and lower associated downstream costs. Under smaller lead-time improvements, ML-enabled screening remained cost-effective in our analyses when precision increased, whereas scenarios with larger lead-time gains tolerated lower precision while still meeting commonly used willingness-to-pay thresholds. This trade-off highlights that the cost-effectiveness of ML-based screening strategies is jointly determined by the magnitude of earlier case identification and the operational performance characteristics of the algorithm, underscoring the importance of tailoring algorithm deployment and threshold selection to real-world screening workflows. Third, because this model was trained using data on patients in ambulatory care in the U.S., it is unclear whether these results can be generalized to other settings or countries. Other work has shown that ML algorithms can be highly effective in identifying HCV in other populations, such as health care workers and children in Egypt [41,42]. Further work assessing cost-effectiveness in other countries and populations is warranted. Fourth, we assumed that patients would be flagged 6.5 months earlier by the algorithm and thus diagnosed and treated 6.5 months earlier. However, it is likely that there would be a delay between getting flagged by the algorithm and a diagnosis and subsequent treatment. To ensure that the ML algorithm findings actually enable clinical change, providers would need to be trained in how to understand and take action based on the algorithm flags. Finally, although the base-case analysis assumed no direct cost associated with the ML algorithm, real-world implementation would likely entail provider training, software integration, and maintenance costs. In sensitivity analyses, the ML-enabled strategy remained cost-effective at the 30% recall level even when a per-person algorithm cost of up to $3 was applied, suggesting that modest implementation costs are unlikely to materially alter conclusions. Nevertheless, future prospective studies should explicitly measure implementation, training, and system-level costs to more fully characterize real-world value. Despite these limitations, our analysis shows that the use of ML algorithms to diagnose HCV patients is a potentially cost-effective method for screening in the United States; further validation data needs to be gathered using prospective data.

## Figures and Tables

**Table 1:** Table of model parameters along with their sampling distributions. * Inflated to 2025 values using the Consumer Price Index for Medical Care. HCV: hepatitis C virus, DC: decompensated cirrhosis, HCC: hepatocellular carcinoma, SVR: sustained viral response.

Parameter	Sampling Distribution and Parameters	Mean Sampled Value (95% Interval)	Source
METAVIR stage fibrosis progression per year (F0–F1, F1–F2, F2–F3, F3–F4)	Not Sampled	0.333	[11]
F4 to DC progression rate per year	Beta (58.49116, 1380.788)	0.0406 (0.0311–0.0522)	[12,13,14]
F4 to HCC progression rate per year	Beta (52.83443, 2417.472)	0.0213 (0.0159–0.0277)	[12,13,14]
DC to HCC progression rate per year	Beta (1.9326, 136.1074)	0.0146 (0.0017–0.0431)	[12]
DC/HCC to liver transplant per year	Beta (1.152814, 36.03474)	0.0302 (0.0012–0.1011)	[12,13,15]
Relative risk of progression after SVR for F4 to DC compared to no SVR	Lognormal (−2.65926, 0.53562)	0.08 (0.02–0.2)	[16]
Relative risk of progression after SVR for F4 to HCC compared to no SVR	Lognormal (−1.469676, 0.214211)	0.24 (0.15–0.37)	[16,17]
HCV spontaneous clearance rate	Uniform (0.22–0.29)	0.25 (0.22–0.29)	[18]
Loss to follow-up rate per year	Uniform (0.4–0.6)	0.5	[4,19]
SVR rate	Not sampled	0.95	[20]
Prevalence of undiagnosed HCV infection newly diagnosed in the 12-month prediction window	Not sampled	0.0002	[9]
HCV antibody prevalence among PWID	Not sampled	0.514	[21,22]
HCV antibody prevalence among 1945–1965 birth cohort	Not sampled	0.016	[23]
HCV antibody prevalence among general population	Not sampled	0.005	[23]
**Mortality Rates**			
F4 per year	Beta (12.44677, 371.1121)	0.0324 (0.01747–0.05275)	[24]
DC per year	Beta (11.61594, 40.93614)	0.218 (0.1196–0.3381)	[24]
HCC per year	Beta (11.61594, 40.93614)	0.220 (0.1216–0.3365)	[24,25]
Transplant year 1	Beta (75.4499, 364.4907)	0.1719 (0.1392–0.2082)	[26]
Transplant year 2+	Beta (97.65551, 2665.93)	0.0354 (0.0289–0.0426)	[26]
General background mortality per year (1/expected lifespan from age 50)	1/30	N/A	[5]
Average time to diagnosis (1/screening rate in years)		6.5 months	[27]
**Annual Costs (2025 $USD)**			
F0–F3 (with or without SVR)	Uniform [1982, 5947]	3939 (2078–5847)	[28] *
F4 (with or without SVR)	Uniform [3518, 10,579]	7114 (3727–10,435)	[28] *
DC (with or without SVR)	Uniform [6722, 20,151]	13,539 (7058–19,766)	[28] *
HCC (with or without SVR)	Uniform [33,625, 100,875]	73,560 (36,020–99,581)	[29,30,31] *
Transplant year 1	Uniform [138,405, 415,217]	278,695 (146,866–409,280)	[31] *
Transplant year 2+	Uniform [33,905, 101,715]	68,425 (35,919–100,052)	[31] *
HCV treatment delivery (per course)	Uniform [767, 2303]	1517 (812–2257)	[32] *
HCV antibody test	N/A	14	Clinical fee schedule
HCV RNA test	N/A	43	Clinical fee schedule
FibroScan	N/A	32	Clinical fee schedule
HCV drug treatment per course	N/A	25,000	[33,34]
**Health Utilities**			
F0	Beta (59.95413, 4.512676)	0.93 (0.86–0.98)	[15,35,36]
F1–F2	Beta (29.92649, 4.871755)	0.86 (0.72–0.95)	[15,35,36]
F3	Beta (12.30437, 2.520171)	0.83 (0.61–0.97)	[15,35,36]
DC	Beta (39.8121, 17.06233)	0.70 (0.57–0.80)	[15,35,36]
HCC	Beta (35.508, 17.48901)	0.67 (0.55–0.78)	[15,35,36]
Post-transplant	Beta (7.612184, 3.109202)	0.71 (0.41, 0.93)	[15,35,36]
Incremental increase in health utilities upon SVR	N/A	0.05	[37]

**Table 2:** Machine learning algorithm cost-effectiveness results.

Algorithm Recall Level	Precision	Accuracy	Cost per Person USD (2.5, 97.5%)	QALYs per Person (2.5–97.5%)	Incremental Cost per Person (2.5, 97.5%)	Incremental QALYs per Person (2.5, 97.5%)	Mean ICER ($/QALY Gained) Compared to Baseline
Baseline (no algorithm)	Baseline (no algorithm	Baseline (no algorithm)	24.79 (17.52–32.00)	16.38 (16.38–16.38)	N/A	N/A	N/A
0.05	0.0202	0.9993	24.63 (17.35–31.83)	16.38 (16.38–16.38)	−0.16 (−0.18–−0.14)	0.0000004 (0.000006, 0.000013)	cost-saving
0.1	0.0112	0.9981	24.83 (17.55–32.04)	16.38 (16.38–16.38)	0.04 (0.004–0.08)	0.000003 (0.000002, 0.000004)	15,298
0.15	0.0066	0.9953	25.09 (17.80–32.30)	16.38 (16.38–16.38)	0.30 (0.23–0.35)	0.000005 (0.000004, 0.000007)	56,079
0.2	0.0047	0.9914	25.36 (18.07–32.59)	16.38 (16.38–16.38)	0.57 (0.48–0.64)	0.000008 (0.000006, 0.000010)	73,078
0.25	0.0036	0.986	25.66 (18.37–32.90)	16.38 (16.38–16.38)	0.87 (0.76–0.96)	0.000010 (0.000008, 0.000012)	84,821
0.3	0.0027	0.9777	25.99 (18.70–33.24)	16.38 (16.38–16.38)	1.20 (1.07–1.31)	0.000013 (0.000010, 0.000016)	94,022
0.35	0.0021	0.9666	26.35 (19.06–33.61)	16.38 (16.38–16.38)	1.56 (1.41–1.68)	0.000015 (0.000012, 0.000019)	102,280
0.4	0.0017	0.9529	26.743 (19.46–34.02)	16.38 (16.38–16.38)	1.95 (1.78–2.10)	0.000018 (0.000014, 0.000022)	110,370
0.45	0.0014	0.9357	28.18 (19.90–34.47)	16.38 (16.38–16.38)	2.40 (2.19–2.55)	0.000020 (0.000016, 0.000025)	118,730
0.5	0.0012	0.9167	27.65 (20.36–34.95)	16.38 (16.38–16.38)	2.86 (2.64–3.03)	0.000023 (0.000018, 0.000029)	126,370

## Data Availability

Any additional data generated from this study are available from the corresponding author upon reasonable request.

## Supplementary Materials

The following supporting information can be downloaded at: https://www.mdpi.com/article/10.3390/v18030299/s1, Table S1: Demographic distribution of HCV-infected patients identified by the ML algorithm by sensitivity level. Table S2: Age group proportions of HCV patients by ML recall proportion. Table S3: Initial fibrosis distributions by ML recall proportion. Table S4: Recall proportion and PPV of the ML Algorithm. Table S5: Simulated fibrosis distributions by age group. Table S6: General Background Mortality by Age.

## Abbreviations

The following abbreviations are used in this manuscript:

DALY	Disability adjusted life-year
HCV	Hepatitis C virus
HCC	Hepatocellular carcinoma
ICER	Incremental cost-effectiveness ratio
PWID	People who inject drugs
US	United States
