# Supplementary material for: Potential Cost-Effectiveness of Machine Learning-Enabled Primary Care Identification of Hepatitis C Virus Patients in the US
Source: Viruses. 2026 Feb 28;18(3):299. doi: 10.3390/v18030299 (PMC13030576; doi:10.3390/v18030299)
Supplement: Supplementary file 1 [file viruses-18-00299-s001.zip › viruses-4112195-supplementary.pdf]

Supplementary Information

Table S1 Demographic distribution of HCV-infected patients identified by the ML algorithm by sensitivity level

ML Algorithm Recall proportion	Proportion who are PWID	Proportion who are in the 1945-1965 'Birth Cohort'	Proportion who are in the general population (non-PWID, non birth cohort)
0.05	0.608	0.271	0.121
0.1	0.604	0.282	0.114
0.15	0.577	0.294	0.129
0.2	0.544	0.315	0.141
0.25	0.497	0.337	0.166
0.3	0.456	0.367	0.177
0.35	0.424	0.392	0.184
0.4	0.398	0.407	0.195
0.45	0.374	0.423	0.203
0.5	0.351	0.436	0.213
0.55	0.329	0.449	0.222
0.6	0.308	0.462	0.23
0.65	0.29	0.473	0.237
0.7	0.274	0.475	0.251
0.75	0.263	0.47	0.267
0.8	0.249	0.473	0.278
0.85	0.238	0.472	0.29
0.9	0.227	0.473	0.3
0.95	0.218	0.474	0.308
1	0.209	0.479	0.312

Table S2: Age group proportions of HCV patients by ML recall proportion

ML recall proportion	Proportion Ages 0-34	Proportion Ages 35-54	Proportion Ages 55+
**0.05**	0.319	0.328	0.353
**0.1**	0.288	0.328	0.384
**0.15**	0.27	0.334	0.396
**0.2**	0.246	0.339	0.415
**0.25**	0.224	0.331	0.445
**0.3**	0.205	0.323	0.472
**0.35**	0.2	0.309	0.491
**0.4**	0.195	0.303	0.502
**0.45**	0.186	0.299	0.515
**0.5**	0.18	0.294	0.526

Table S3: Initial fibrosis distributions by ML recall proportion

ML recall proportion	F0	F1	F2	F3	F4
**0.05**	0.1726822	0.1685953	0.1372957	0.1320529	0.3893739
**0.1**	0.1576472	0.1580584	0.1346576	0.1343376	0.4152992
**0.15**	0.1492166	0.1528396	0.1343834	0.1366056	0.4269548
**0.2**	0.1378261	0.1454315	0.133389	0.1391589	0.4441945
**0.25**	0.1267569	0.1367545	0.12984	0.1395251	0.4671235
**0.3**	0.1171427	0.1290972	0.1265463	0.1396702	0.4875436
**0.35**	0.1140191	0.1252991	0.1231864	0.1378421	0.4996533
**0.4**	0.1112947	0.1227002	0.1215033	0.1372692	0.5072326
**0.45**	0.1067301	0.1190415	0.119899	0.1373049	0.5170245
**0.5**	0.1035706	0.1162526	0.1183404	0.1369626	0.5248738

Table S4: Recall proportion and PPV of the ML Algorithm

Recall proportion	Positive Predictive Value
0.05	0.02020028
0.1	0.011196891
0.15	0.006553752
0.2	0.004723737
0.25	0.003589376
0.3	0.002706939
0.35	0.00205348
0.4	0.001695035
0.45	0.001394058
0.5	0.001200212
0.55	0.001009942
0.6	0.000869559
0.65	0.00076098
0.7	0.000655526
0.75	0.00057

0.8	0.000480978
0.85	0.000403186
0.9	0.000350131
0.95	0.000296159
1	0.000218388

Table S5: Simulated fibrosis distributions by age group

Age Group	F0	F1	F2	F3	F4
Ages 0-34	0.4866	0.3509	0.1265	0.0304	0.0056
Ages 35-54	0.0515	0.1609	0.251	0.261	0.2756
Ages 55+	0.0016	0.011	0.0414	0.1041	0.8419

Table S6: General Background Mortality by Age:

Age Range	Mortality Rate
50-54	0.005
55-59	0.008
60-64	0.011
65-69	0.015
70-74	0.023
75-79	0.036
80-84	0.058
85+	0.14
